# Impact of Glycine–Serine Linker on Target Antigen Binding and Subsequent CD37CAR-T Performance

**DOI:** 10.3390/ijms27094112

**Published:** 2026-05-04

**Authors:** Wannakorn Khopanlert, Napat Prompat, Jirakrit Saetang, Kajornkiat Maneechai, Shingo Okuno, Seitaro Terakura, Jakrawadee Julamanee

**Affiliations:** 1 Stem Cell Laboratory, Stem Cell Transplantation and Cellular Therapy Excellence Center, Songklanagarind Hospital, Faculty of Medicine, Prince of Songkla University, Hat Yai 90110, Songkhla, Thailand; wannakorn.k@psu.ac.th (W.K.);; 2 Anatomical Pathology Unit, Division of Pathology, Faculty of Medicine, Prince of Songkla University, Hat Yai 90110, Songkhla, Thailand; 3 Thailand Hub of Talents in Cancer Immunotherapy (TTCI), Bangkok 10330, Thailand; 4 Faculty of Medical Technology, Prince of Songkla University, Hat Yai 90110, Songkhla, Thailand; 5 International Center of Excellence in Seafood Science and Innovation, Faculty of Agro-Industry, Prince of Songkla University, Hat Yai 90110, Songkhla, Thailand; 6 Department of Hematology and Oncology, Nagoya University Graduate School of Medicine, Nagoya 466-8560, Japan; 7 Hematology Unit, Division of Internal Medicine, Faculty of Medicine, Prince of Songkla University, Hat Yai 90110, Songkhla, Thailand

**Keywords:** linker alteration, GS4 linker, CD37, chimeric antigen receptor, CD28/CD40

## Abstract

CD19-chimeric antigen receptor (CAR) has shown promising outcomes in B-cell malignancies. However, relapses due to poor CAR-T persistence and antigen escape have occurred. CD37 is a potential alternative immunotherapy for CD19^−^ tumors. Inferior CAR-T cytotoxicity was observed in CD37CAR-T compared to CD19CAR-T model that was possibly due to lower CD37CAR affinity. To alleviate CD37CAR functions, we optimized the most prevalent linkers, Whitlow (18aaL) and glycine–serine (GS4L). CD37.GS4L CAR-T showed higher transduction efficiency and T-cell expansion contributed by minimizing T-cell fratricide. In chronic antigen stimulation, CD37.GS4L CAR demonstrated robust T-cell proliferation while preserving stemness and a decrease in induced exhaustion phenotype, which resulted in greater tumoricidal activity among various CD37^+^ malignancies. In silico analysis showed that CD37.GS4L scFv altered structural dynamic behaviors by facilitating variable heavy-chain region closer to CD37 receptor with higher binding affinity and less aggregation of negatively charged protein, which contributed to lower tonic signaling during CAR activation and diminished exhaustion. Ultimately, effective anti-tumor control with notable memory T-cell persistence was exhibited in Burkitt lymphoma mice treated with CD37.GS4L CAR-T. Additionally, CD37.GS4L CAR-T illustrated the potential in vivo cytotoxicity in myeloma-inoculated mice. In summary, the flexibility and affinity of glycine–serine linker of CD37CAR can potentiate CAR-T functionality.

## 1. Introduction

Adoptive cell therapy with T cells genetically modified to express CD19-chimeric antigen receptor (CD19CAR) incorporating CD28 or 4-1BB as a co-stimulatory domain has shown encouraging outcomes in B-cell malignancies [1,2,3,4,5,6,7,8,9,10]. However, patients have encountered relapses attributed to insufficient CAR-T persistence and antigen escape [1,2,3,4,5,6]. The CAR structural designs have been studied to address these issues, including ligand binding, spacer, transmembrane, and cytoplasmic domain modification [11,12,13,14,15,16].

CD37 is a four-passage transmembrane protein of the tetraspanin superfamily, which is a potential target for immunotherapy as its expression is mainly restricted to mature B-cell malignancies with some expression in T-cell malignancies including cutaneous T-cell lymphoma, peripheral T-cell lymphoma (PTCL), and some solid tumors such as glioma, neuroblastoma, lung, and liver malignancies [17,18,19,20]. Recently, CD37 mRNA and protein were discovered in acute myeloid leukemia samples and showed a prognostic correlation [21,22]. Several studies have confirmed the successful generation of CD37CAR-T cells incorporating either CD28 or 4-1BB against CD37^+^ lymphoid malignancies as an alternative treatment in CD19^−^ lymphoid tumors [18,23,24,25]. Unfortunately, the latest clinical phase I CD37CAR-T study failed to demonstrate survival advantages in five T-cell lymphoma patients, which was attributed to cytokine driven severe pancytopenia and infection [26].

To potentiate CAR function, previous studies have explored how CAR hinge and spacer affect CAR-T efficiency [13,14,25]. Okuno et al. demonstrated that optimizing short 15 amino acid spacers in CD37CAR-T cells preserves anti-tumor activity while minimizing self-ligation-associated CAR-T cell fratricide, with minimal impact on normal hematopoietic cells [25]. In addition, intracellular signaling molecules also play an important role in potentiating CAR-T function and persistence. Our group developed the dual T-/B-cell co-stimulatory molecule, CD28/CD40, to synergize both canonical and non-canonical NF-κB signaling of CAR-T cells. CD19.28.40z CAR-T cells showed incremental basal NF-κB signaling and memory gene signatures that maximized CAR-T cell proliferation, persistence, and anti-tumor efficacy both in vitro and in murine xenograft models. CD37CAR incorporating the CD28/CD40 signaling domain further showed superior CAR-T proliferation, cytotoxicity, and diminished expression of T-cell exhaustion markers compared to CD28 and 4-1BB [15]. However, inferior CAR-T cytotoxicity was observed in the CD37CAR-T compared to the CD19CAR-T model that was possibly due to the lower affinity of anti-CD37 single-chain variable fragment (scFv) [15,25]. Additionally, Singh et al. revealed that the shorter length of the glycine–serine scFv linker influenced CD22CAR-T affinity and function [27]. Nevertheless, direct comparisons among the scFv linkers have not been investigated.

In the present study, we compared the most prevalent linkers GSTSGSGKPGSGSGSTKG (Whitlow, 18 amino acid linker; 18aaL) [25,28] with GGGGSGGGGSGGGGSGGGGS (glycine–serine; GS4L) [24,29] in terms of regulating CD37CAR-T effectiveness against various CD37^+^ malignancies. 

## 2. Results

### 2.1. CD37 Expression Across Various Types of Tumors

We explored CD37 mRNA expressions using the Cancer Cell Line Encyclopedia to assess the potential targets for CD37CAR-T cell among various types of tumors and found restricted expression on mature B-cell malignancies, PTCL, and some solid tumors [17,18,19,20]. We then examined the expression of CD37 on multiple cell lines and found the highest level of expression was on lymphoma cell lines that included Raji (Burkitt lymphoma), Ramos (Burkitt lymphoma), Jeko-1 (mantle cell lymphoma), and solid tumor cell lines, SNU449 (hepatocellular carcinoma) and NCI-H522 (adenocarcinoma of lung) were in the positive range of 99.6–100%. Moderate CD37 expression was observed on HUT78 (Sezary syndrome), MM.1S (multiple myeloma), and KMS-12-BM (multiple myeloma) that showed 45.8–73.6% positivity, while K562 (chronic myeloid leukemia) did not express CD37 (Figure S1A). We designated Raji cell line as the primary target for downstream experiments to further investigate CD37CAR-T cell performance.

### 2.2. GS4L Linker Modification on CD37CAR Altered Gene Transfer and Functions

**Figure 1:**
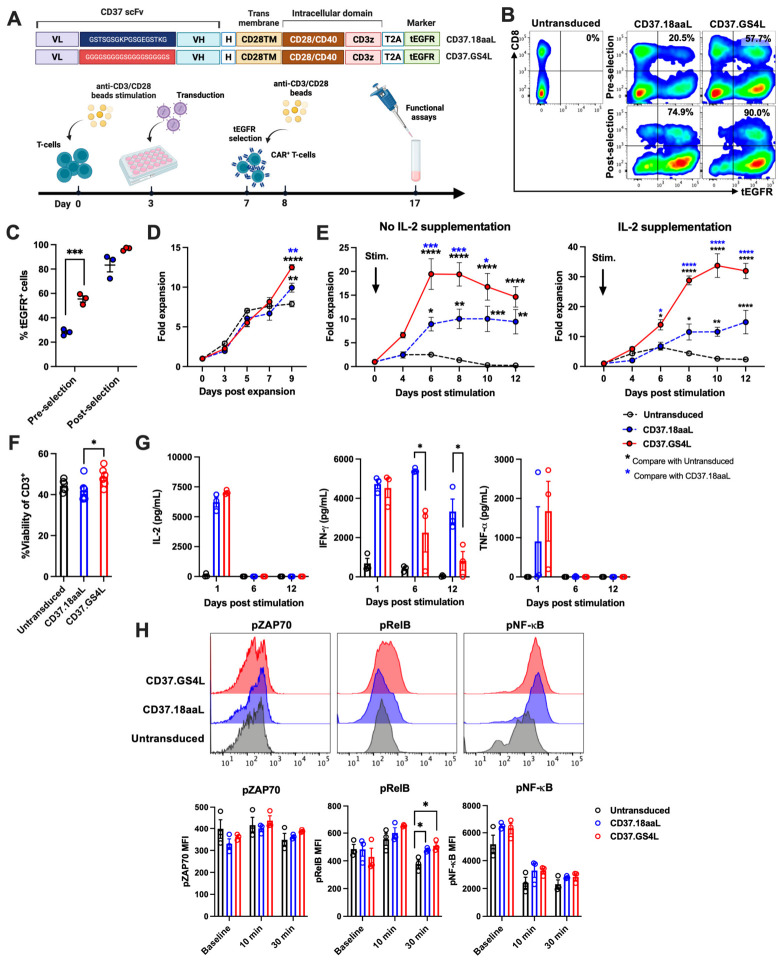
**CD37 chimeric antigen receptor (CAR) T-cell generation.** (**A**) Illustration of the third-generation of CD37CAR constructs with CAR-T cell generation. Two different linkers (GSTSGSGKPGSGEGSTKG [Whitlow, 18aaL] and GGGGSGGGGSGGGGSGGGGS [glycine–serine, GS4L]) were fused into the anti-CD37CAR backbone (CD37scFv) VL–linker–VH–H–CD28TM–CD28/CD40IC–CD3ζ–tEGFR. scFv, single-chain variable fragment; VL, light-chain variable fragment; VH, heavy-chain variable fragment; H, short 12 amino acids of IgG4 Fc-derived spacer of hinge; TM, transmembrane domain; IC, intracellular domain; tEGFR, truncated EGFR. (**B**) Representative flow plots of CD37CAR-T identified by tEGFR staining at pre- and post-selection. (**C**) CD37CAR-T cell transduction efficacy and enrichment. (**D**) CD37CAR-T cell expansion after stimulation with anti-CD3/CD28 Dynabeads. (**E**) Proliferation assays. Untransduced-T or CD37CAR-T cells were stimulated with γ-irradiated Raji cells at 1:2 ratio and cultured without (**left**) or with (**right**) exogenous IL-2. The viable T-cells were counted for 12 days. Arrows mark the day of being stimulated with Raji cells. (**F**) Autologous T-cell fratricide. Untransduced-T or CD37CAR-T were cultured with autologous T-cell at 1:1 ratio for 24 h and assessed for viability. (**G**) Measurement of IL-2, IFN-γ, and TNF-α concentrations. Culture medium in T-cell proliferation assay was collected and measured with ELISA on days 1, 6, and 12. (**H**) Representative flow plots of intracellular phospho-flow analysis of phospho-ZAP70 (pZAP70), phospho-RelB (pRelB), and phospho-NF-κB (pNF-κB) of untransduced-T or CD37CAR-T after Raji cell stimulation at a 1:5 ratio for 30 min (**upper**) and the pool data at baseline and after 10 and 30 min of T-cell stimulation (**lower**). Data were pooled from three different donors and presented as mean ± SEM. Student’s *t*-test for (**C**), Two-way ANOVA for (**D**,**E**), and one-way ANOVA for (**F**–**H**); * *p* < 0.05, ** *p* < 0.01, *** *p* < 0.001, **** *p* < 0.0001.

We cloned the two different scFv linkers, GSTSGSGKPGSGEGSTKG (Whitlow, 18aaL) [28] and GGGGSGGGGSGGGGSGGGGS (GS4L), on the third-generation CD37CAR endowed CD28/CD40 co-stimulatory domain backbone [15] to examine the influence of linkers on CAR-T functions. The CD37CAR constructs and CAR-T generation are described in Figure 1A. Both CD37CAR genes were successfully transferred into primary CD3^+^ cells with significantly higher transduction efficacy in CD37.GS4L 55.4% (range: 50.8–59.4%) than CD37.18aaL 28.1% (range: 25.6–30.6%) and evenly purified for over 96.5% (range: 95.0–97.1%) in CD37.GS4L compared to 83.3% (range: 72.2–90.0%) in CD37.18aaL (Figure 1B,C). The enriched CD37CAR-T were then expanded for subsequent assays using anti-CD3/CD28 Dynabeads. The CD37.GS4L CAR-T notably showed higher fold expansion compared to CD37.18aaL CAR-T and untransduced-T cells (Figure 1D). Furthermore, proliferation assays initially confirmed the robust proliferative capacity of CD37.GS4L CAR-T cells after stimulation with CD37^+^ target cells regardless of cytokine supplementation compared to CD37.18aaL CAR-T (Figure 1E). We hypothesized that the inferior transduction efficacy and proliferative capacity of CD37.18aaL CAR-T cells would be related to autologous T-cell killing or fratricide. To assess such phenomenon, untransduced-T or CD37CAR-T cells were co-cultured overnight with autologous CD3^+^ cells where CD37.18aaL exhibited significant T-cell fratricide compared to CD37.GS4L (Figure 1F). Moreover, CD37.18aaL consistently secreted interferon-γ (IFN-γ) throughout the culture period, which was attributed to the lower proliferative capacity results (Figure 1G). On the contrary, interleukin-2 (IL-2) and tumor necrosis factor-α (TNF-α) were evenly secreted by CD37.18aaL CAR-T and CD37.GS4L CAR-T cells during the early phase following target cell stimulation (Figure 1G). Additionally, the intracellular cytokine secretion assay for IL-2, IFN-γ, Granzyme B, and perforin-positive cells following short-term antigen stimulation showed insignificant differences among CAR-T cells (Figure S1B). We performed intracellular phospho-flow analysis to further assess the signaling alteration following CD37 antigen stimulation. At baseline, there was no significant increment in tonic signaling among CD37.GS4L CAR-T or CD37.18aaL CAR-T cells (Figure 1H). However, CD37.GS4L CAR-T exhibited a trend of higher phospho-ZAP70 (pZAP70) and phospho-RelB (pRelB) compared to CD37.18aaL CAR-T after activation for 30 min (Figure 1H). These results indicate the impact of scFV linkers on CAR-T expression and functions, especially CAR-T cell fratricide, proliferative capacity, and cytokine secretion.

### 2.3. GS4L Linker Sustained CD37CAR-T Cell Proliferation While Preserving Stemness with a Reduction in Induced Exhaustion

**Figure 2:**
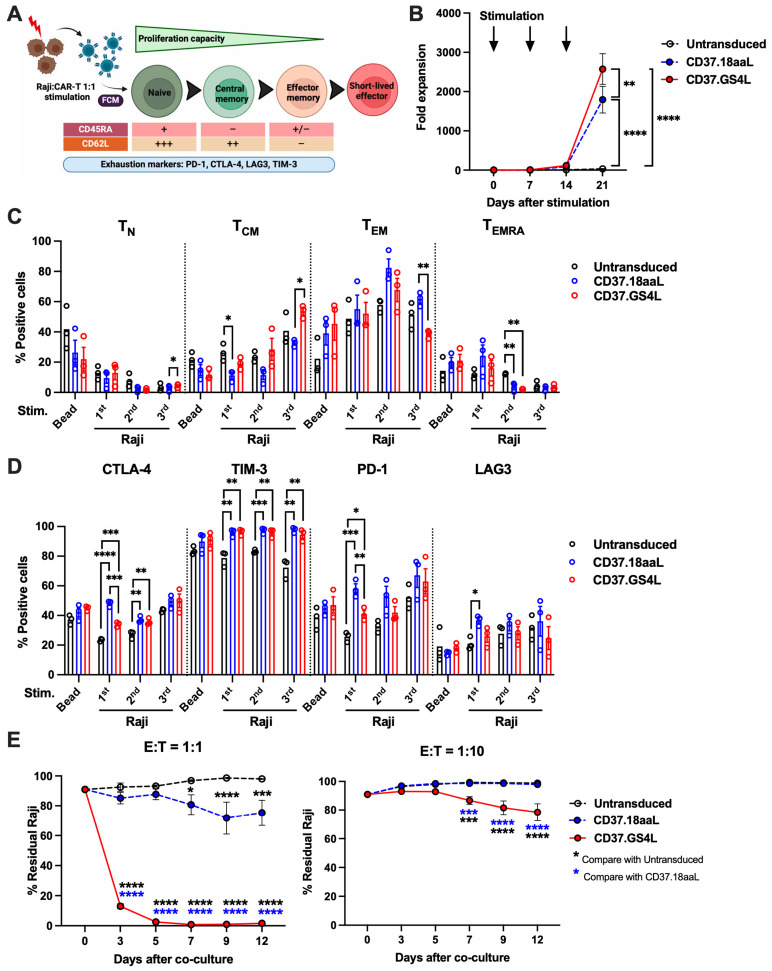
**Chronic antigen stimulation assay.** (**A**) Illustration of chronic antigen stimulation and immunophenotype assays. Untransduced-T or CD37CAR-T cells were stimulated weekly with γ-irradiated Raji cells for three consecutive weeks at a 1:1 ratio and cultured with IL-2 supplemented medium. T-cell differentiation subsets are classified into CD45RA^+^/CD62L^+^ naïve T (T_N_), CD45RA^−^/CD62L^+^ central memory T (T_CM_), CD45RA^−^/CD62L^−^ effector memory T (T_EM_), and CD45RA^+^/CD62L^−^ effector memory re-expressing CD45RA T (T_EMRA)_. (**B**) T-cell fold expansion. Arrows mark the day of Raji cell stimulation. (**C**) T-cell differentiation subsets. (**D**) T-cell exhaustion phenotypes. (**E**) Prolonged co-culture assay. Untransduced-T or CD37CAR-T cells were co-cultured with Raji/ffluc-GFP at E:T ratios of 1:1 (**left**) and 1:10 (**right**) for 12 days without IL-2 supplementation. The residual tumor cells were assessed by flow cytometry at indicated time points. Data were pooled from three different donors and shown as mean ± SEM; Two-way ANOVA for (**B**,**E**); one-way ANOVA for (**C**,**D**); * *p* < 0.05, ** *p* < 0.01, *** *p* < 0.001, **** *p* < 0.0001.

We performed chronic antigen stimulation assay to simulate chronic antigen stimulation in vivo by co-culturing untransduced-T or CD37CAR-T cells with γ-irradiated Raji cells for three consecutive weeks to replicate the effects of prolonged antigen exposure in humans. The differences in T-cell expansion, differentiation subsets, and exhaustion phenotypes at baseline and post-stimulation among CD37CAR-T constructs were evaluated over time (Figure 2A). CD37.GS4L CAR-T cells revealed substantial expansion (Figure 2B) with significant preservation of CD45RA^+^/CD62L^+^ naïve T (T_N_) and CD45RA^−^/CD62L^+^ central memory T (T_CM_) (Figure 2C) along with reduced expression of exhaustion phenotypes, especially CTLA-4 and PD-1 (Figure 2D) compared with CD37.18aaL CAR-T. We then validated the superior CD37.GS4L CAR-T cell function in prolonged co-culture assay to confirm the potent anti-tumor eradication over CD37.18aaL CAR-T. Notably, CD37.GS4L CAR-T continually suppressed tumor cell growth throughout the co-culture period at effector to target (E:T) ratios of 1:1 and 1:10 compared to CD37.18aal CAR-T, regardless of the IL-2 effect (Figure 2E). These results highlight the functional advantages of CD37.GS4L CAR in T-cell proliferation, stemness, and exhaustion, which translated into greater anti-tumor cytotoxicity compared to CD37.18aaL CAR.

### 2.4. In Silico Study Reveals That Linkers Influence the Functionality of CD37CAR-T Cells

**Figure 3:**
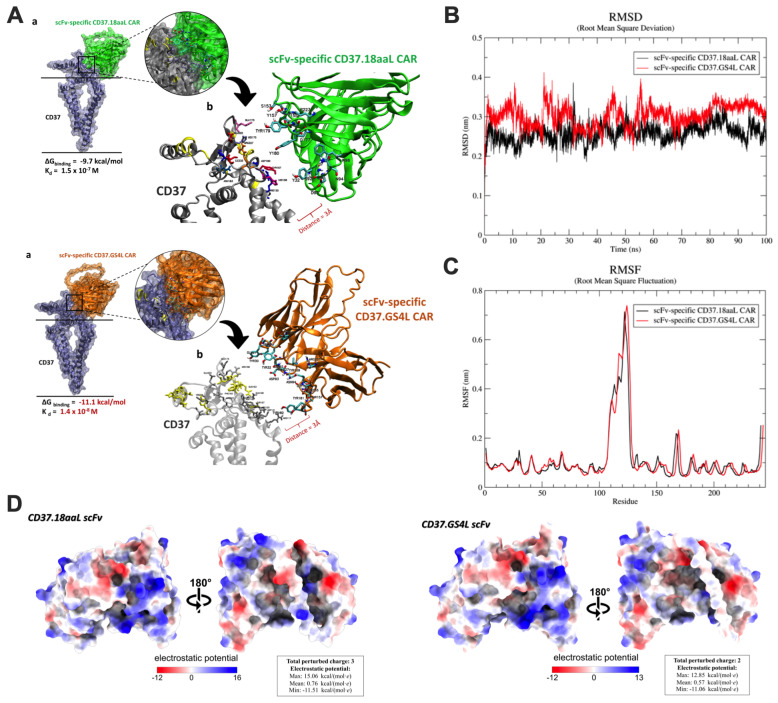
**In silico study of CD37CAR-T cells.** (**A**) The structure of CD37.18aaL (**upper**) and CD37.GS4L (**lower**) single-chain variable fragment (scFv). (**B**) Root-mean-square deviation (RMSD) of scFv-specific CD37CAR. (**C**) Root-mean-square fluctuation (RMSF) during 100 ns molecular dynamic (MD) simulation of scFv-specific CD37CAR. (**D**) Electrostatic surface potential of the three-dimensional homology models for CD37.18aaL (**left**) and CD37.GS4L (**right**) CAR scFv. Total perturbed charge calculation of the structures was carried out via the AMBER ff99SB protein forcefield. The electrostatic surface was determined with the default “*coulombic*” command in ChimeraX.

We further investigated the effects of the linker region on CD37 scFv toward its structural behaviors and the binding characteristics of CD37 at the atomistic level. The predicted 3D structure of both CD37.18aaL and CD37.GS4L scFv was built based on de novo folding that was guided by deep machine learning (Figure 3A). Molecular dynamic (MD) simulation was employed to elucidate the dynamic behaviors of these modeled structures through 100 nanoseconds (ns) of simulation time. The predicted structures demonstrated conformation stability according to the root-mean-square deviation (RMSD) value of 2.27 and 2.97 Å in CD37.18aaL and CD37.GS4L scFv, respectively (Figure 3B). Stable patterns of radius of gyration in the two models were observed throughout the simulation, which indicated less effect on the overall conformation and folding of the proteins. Apart from conformational stability, the flexibility of antibodies was investigated through root-mean-square fluctuation (RMSF) plots (Figure 3C). CD37.GS4L scFv revealed more flexibility at residues 108–127 (linker-loop region), 150–160, and 200–210 (VH region) compared to CD37.18aaL. Since the VH region is responsible for receptor binding and facilitating immune responses, an incremental change in the flexibility of these regions may affect the biological activity of CAR. In addition, protein–protein docking was applied to the 3D structural modeling of both scFv against the human CD37 receptor. The results revealed that the CD37.GS4L scFv had better binding affinity with a predicted ∆G_binding_ value of −11.1 kcal/mol and dissociation constant (K_d_) of 1.4 × 10^−8^ molar, whereas the scFv specific CD37.18aaL yielded a predicted ∆G_binding_ value of −9.7 kcal/mol and K_d_ of 1.5 × 10^−7^ molar (Figure 3A). 

To quantify the potential electrostatic properties on each CAR surface, we calculated the surface electrostatic characteristics of CD37.18aaL and CD37.GS4L scFv, which demonstrated that the electrostatic potential (EPS) ranged from −11.51 to +15.06 kcal/(mol·e) and −11.06 to +12.85 kcal/(mol·e), respectively (Figure 3D). The coloring scheme displayed the negative (red), neutral (white), and positive (blue) potentials that were regionally distributed on the molecular surfaces of CD37.CAR scFvs. Furthermore, CD37.18aaL scFv had a total perturbed charge of 3, while CD37.GS4L exhibited a total perturbed charge of 2, which indicated a lower electrostatic profile (Figure 3D).

Taken together, CD37.GS4L scFv altered the structural dynamic behaviors by facilitating the VH region closer to the CD37 receptor with higher binding affinity and less aggregation of the negatively charged protein, which contributed to the improvement in its functionality.

### 2.5. CD37.GS4L CAR-T Demonstrated Potent Tumoricidal Activity in Burkitt’s Lymphoma Xenograft Model

**Figure 4:**
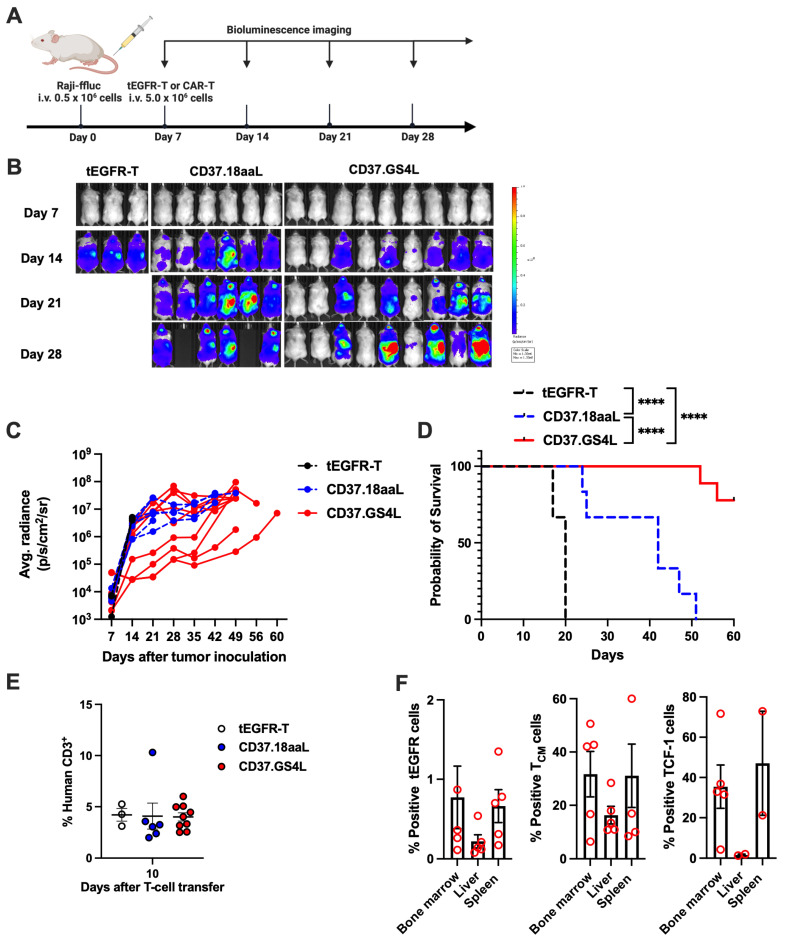
**Potent cytotoxicity of CD37.GS4L CAR-T cells in Burkitt’s lymphoma model.** (**A**) Schematic of the in vivo Burkitt’s lymphoma murine experiment. NOD/Shi-scid common-γ chain knockout (NSG) mice were intravenously injected with Raji/ffluc-GFP (0.5 × 10^6^ cells) via the tail vein on day 0. Mice were then separately treated with tEGFR-T or CD37CAR-T (5.0 × 10^6^ cells) on day 7. Tumor burden was assessed at indicated time points. (**B**) Representative of tumor volume measured by bioluminescence imaging (BLI) of Raji-inoculated NSG mice over time. (**C**) Tumor burden of individual mice treated with tEGFR-T or CD37CAR-T cells that was calculated and reported as average radiance, which is the sum of the radiance from each pixel inside the region of interest divided by the number of pixels or superpixels. Data were pooled from two independent experiments using three different donors (CD37CAR-T cells: *n* = 6–9 per group; tEGFR-T: *n* = 3). (**D**) Kaplan–Meier survival analysis of Raji-inoculated NSG (Log-rank (Mantel–Cox) test; **** *p* < 0.0001). (**E**) Percentage of human T-cells in murine peripheral blood after T-cell transfer for 10 days; one-way ANOVA; *p* = ns. (**F**) Infiltrative CD45RA^−^/CD62L^+^ central memory (T_CM_) and TCF-1^+^ CAR-T (tEGFR^+^) cells in bone marrow, liver, and spleen of sacrificed mice on day 60 (CD37.GS4L CAR-T: *n* = 2–5).

We next examined the in vivo anti-tumor cytotoxicity using the Raji-bearing NOD/Shi-scid common-γ chain knockout (NSG) mice model. NSG mice were inoculated with Raji/ffluc-GFP (0.5 × 10^6^ cells) via the tail vein and then treated with tEGFR-T or CD37CAR-T (5.0 × 10^6^ cells) on day 7 (Figure 4A). Mice treated with CD37.GS4L CAR-T showed greater tumor clearance with inferior tumor burden (Figure 4B,C), which contributed to prolonged overall survival compared to the control arms (Figure 4D). Furthermore, we assessed human CD3^+^ cells in murine peripheral blood on day 10 after T-cell transfer that showed insignificant difference among the CAR-T constructs (Figure 4E). The mice treated with CD37.GS4L CAR-T and survived were eventually sacrificed on day 60 to evaluate CAR-T persistence and stemness in the bone marrow, liver, and spleen. Interestingly, notable numbers of tEGFR^+^CD37.GS4L CAR-T cells expressing central memory and TCF-1 phenotypes were observed to have infiltrated among the collected reticuloendothelial organs (Figure 4F). Overall, CD37.GS4L CAR-T highlighted the potent anti-tumor cytotoxicity and T-cell persistence in Burkitt’s lymphoma xenograft model.

### 2.6. CD37CAR-T Showed Anti-Tumor Performance on Various CD37^+^ Malignancies

**Figure 5:**
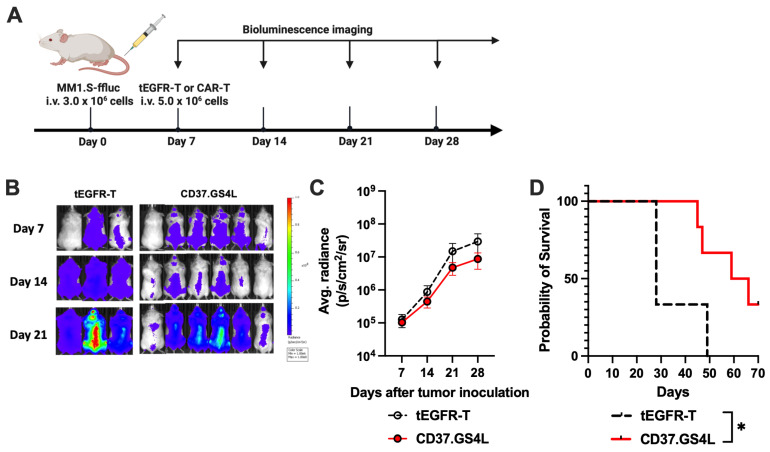
**CD37CAR-T tumoricidal activities against myeloma.** (**A**) Schematic of the in vivo myeloma and T-cell lymphoma murine experiments. NOD/Shi-scid common-γ chain knockout (NSG) mice were intravenously injected with MM.1S/ffluc-GFP (3.0 × 10^6^ cells) on day 0. Mice were then separately treated with tEGFR-T or CD37.GS4L CAR-T (5.0 × 10^6^ cells) on day 7. Tumor burden was assessed at the indicated time points. (**B**) Representative tumor volume was measured by bioluminescence imaging (BLI) of the MM.1S-inoculated NSG mice over time. (**C**) Tumor burden of the mice treated with tEGFR-T or CD37.GS4L CAR-T cells was calculated and reported as average radiance, which was the sum of the radiance from each pixel inside the region of interest divided by the number of pixels or superpixels. Data are presented as mean ± SEM and summarized from three different donors (CD37.GS4L CAR-T cells: *n* = 6; tEGFR-T: *n* = 3); two-way ANOVA; *p* = ns. (**D**) Kaplan–Meier survival analysis of MM.1S-inoculated NSG (log-rank (Mantel–Cox) test; * *p* < 0.05).

The CD37 antigen can be detected on various cell lines including solid tumors (Figure S1A). Therefore, we assessed the anti-tumor capacity of CD37CAR-T against various types of tumors to identify the potential target for CD37CAR-T cell therapy. Untransduced-T or CD37CAR-T cells were co-cultured for 24 h with various cell lines that included K562, Ramos, Jeko-1, HUT78, MM.1S, KMS-12-BM, SNU449, NCI-H522, and two patient-derived primary tumor cells, mantle cell lymphoma (MCL) and chronic lymphocytic leukemia (CLL), which were then assessed for specific cytolysis. Interestingly, CD37.GS4L CAR-T demonstrated higher specific cytolysis in all CD37^+^ tumor cell lines and primary cells except in the negative control (K562) at all E:T ratios compared to the control (Figures S1A and S2A). We adopted two target cell lines with the best cytolytic response, MM.1S and HUT78, for further testing in prolonged co-culture assay. Both CD37CAR-T linkers comparably suppressed tumor cells at the E:T ratio of 1:10 after culture for 12 days (Figure S2B,C). In vivo CAR-T cytotoxicity was evaluated using MM.1S-inoculated NSG mice (Figure 5A). CD37.GS4L CAR-T cells suppressed MM.1S tumor cell growth (Figure 5B,C) that led to significant longer overall survival compared to tEGFR-T (Figure 5D). From these findings, CD37CAR-T cells may be feasible as an alternative therapeutic approach for various types of malignancies.

## 3. Discussion

The utilization of CD37 as a tumor target has shown safety profiles for a CAR-T cell therapeutic approach in hematologic malignancies, both in preclinical and clinical studies [18,23,24,25,26,30,31]. However, previous direct comparisons indicated that CD37-directed CAR-T cells exhibited inferior cytotoxic potency relative to the CD19CAR-T construct [25,31]. The disparity is likely attributable to the lower binding affinity of the employed CD37 scFv due to the influence of the CAR hinge and spacer domains on overall CAR functionality as elucidated by Okuno et al [25]. Singh et al., meanwhile, demonstrated that a shortened glycine–serine linker adversely affects the affinity and therapeutic efficacy of CD22-targeted CAR-T cells [27].

In the current study, we examined whether the scFv linker would affect the distinct functionality of CD37CAR. From previous reports, the two scFv linker sequences, GSTSGSGKPGSGSGSTKG (Whitlow, 18aaL) and GGGGSGGGGSGGGGSGGGGS (glycine–serine, GS4L), were widely utilized within the context of CD37CAR constructs [24,25,26]. CD37CAR that incorporated CD28/CD40 co-stimulatory domains with two different scFv linkers, namely CD37.GS4L and CD37.18aaL, were established to determine their impacts on CAR-T functions against CD37-expressing malignancies [15]. As a proof of concept, the CD37.GS4L CAR-T demonstrated a notable enhancement in the gene transfer process and superior expansion of CAR-expressing T-cells that contributed to the reduction in autologous T-cell fratricide compared to the CD37.18aaL. In terms of signaling transduction, CD37.GS4L CAR-T displayed a trend of incremental expression of pZAP70 and pRelB following CD37 antigen stimulation compared to the 18aaL linker. The observed functional differences arise from linker-dependent modulation of upstream antigen engagement, which likely influences the quality or magnitude of proximal signaling initiation. Linker optimization may enhance immune synapse formation or receptor-triggering efficiency, thereby augmenting signaling output through shared canonical and non-canonical NF-κB pathways. These observations are conceptually consistent with findings by Ajmal et al. and Farooq et al., which demonstrate that enhanced signaling through the ZAP-70/NF-κB axis supports metabolic fitness, stemness, and durable CAR-T persistence [32,33].

The robust CAR-T cell proliferative response upon antigen-specific stimulation was also evidenced in CD37.GS4L CAR-T, which were possibly affected by reduced IFN-γ secretion during the culture. Overactivated CAR-T cells excessively secrete cytokines, especially IFN-γ, which may upregulate inhibitory receptors such as PD-1 and CTLA-4, leading to activation-induced cell death [34,35].Furthermore, the chronic antigen stimulation assay demonstrated sustained proliferation of CD37.GS4L CAR-T cells, which were accompanied by a higher proportion of naïve and central memory T-cell phenotypes and reduced expression of T-cell exhaustion markers, specifically CTLA-4 and PD-1, compared with CD37.18aaL CAR-T cells. These features correlated with improved anti-tumor performance in extended co-culture experiments against various CD37^+^ malignant cells. CD37.18aaL CAR-T cells exhibited impaired serial killing capacity and proliferative fitness, contributing to the marked loss of tumor control under higher antigen burden. By contrast, the GS4L construct maintained a more favorable memory phenotype and lower exhaustion, which likely supported continued expansion and sustained cytotoxicity even at low effector-to-target ratios.

In silico structural modeling was used to further investigate the impact of linker composition on CD37 scFv conformation and its antigen-binding properties at the atomic level. Our analysis revealed that the GS4L linker induced favorable structural rearrangements that brought the VH domain into closer spatial alignment with the CD37 epitope. This configuration was associated with increased binding affinity and reduced aggregation of negatively charged surface residues. The electrostatic refinements are likely to enhance receptor stability and functionality. Importantly, the surface charge distribution analysis indicated that CD37.GS4L CAR exhibited lower densities of positively charged patches (PCPs), which is a feature previously associated with diminished tonic signaling and reduced T-cell exhaustion. These findings align with earlier reports demonstrating that fine-tuning electrostatic interactions within CAR structures can substantially influence signaling thresholds, persistence, and overall therapeutic efficacy [36,37]. Thus, our study provides mechanistic insight into how linker engineering can be strategically leveraged to improve CAR-T cell performance by modulating both structural orientation and surface charge characteristics. Nevertheless, the direct long-term biophysical studies specifically assessing aggregation kinetics would further strengthen this conclusion and represent an important direction for future investigation.

The aforementioned structural and functional enhancements conferred by the GS4L linker significantly improved the in vivo performance of CD37-targeted CAR-T cells. CD37.GS4L CAR exhibited sustained tumor suppression in the CD37^+^ Burkitt’s lymphoma xenograft model that was accompanied by the persistence of infiltrative central memory and TCF-1^+^ CAR-T populations in reticuloendothelial organs. These features correlated with the durable anti-tumor responses and resulted in extended survival in CD37.GS4L-treated mice that far surpassed the outcomes seen in CD37.18aaL CAR or control tEGFR-T. Moreover, this study identified CD37 expression across a range of hematologic and solid malignancies. Thereby, we explored the therapeutic applicability of CD37.GS4L CAR-T that showed effective tumor control and improved survival outcomes compared to tEGFR-T in multiple myeloma-inoculated xenograft models. Nonetheless, the broad activity of CD37.GS4L CAR-T cells across multiple tumor models highlight their promise as a versatile therapeutic platform.

This study has a limitation in that direct assessment of CAR surface expression using CAR- or linker-specific antibodies was not performed. However, the present study employed CAR–T2A–tEGFR backbone constructs, in which tEGFR was designated as a surrogate marker for CAR surface expression. Moreover, previous studies have demonstrated that CAR-transduced T cells exhibit equivalent levels of CAR and tEGFR expression [15,38,39]. The present study was designed as a mechanistic, preclinical investigation rather than a confirmatory hypothesis-testing study. Consequently, a formal prospective power analysis was not performed. Future confirmatory studies using prospectively powered designs will be essential to validate the magnitude and reproducibility of the observed effects.

In summary, the considerable flexibility and affinity of CD37.GS4L scFv potentiate CAR-T functions in terms of proliferation, persistence, and tumoricidal activity in both in vitro and in vivo xenograft models. The glycine–serine linker modification could facilitate scFv activity and maximize CAR-T effectiveness. 

## 4. Material and Methods

### 4.1. Cell Lines

Human cell lines K562, Raji, Ramos, Jeko-1, MM.1S, and KMS-12-BM were maintained in our laboratory. HUT78, SNU449, and NCI-H522 cell lines were purchased from American Type Culture Collection (ATCC, Manassas, VA, USA). Raji, MM.1S, and HUT78 cells expressing green fluorescent protein (GFP) firefly luciferase (ffluc) were lentivirally transduced with the GFP-ffluc gene, according to previous established researches [14,25]. K562, Raji, Ramos, MM.1S, SNU449, and NCI-H522 were cultured in RPMI-1640 (Gibco, Life Technologies Corp., Carlsbad, CA, USA) with 10% fetal bovine serum (FBS), 1% penicillin–streptomycin (PS), and 0.8 mM L-glutamine (LG). Jeko-1 and KMS-12-BM were maintained in RPMI-1640 (Gibco, Life Technologies Corp., CA, USA) supplemented with 20% FBS, 1% PS, and 0.8 mM LG. HUT78 was cultured with Iscove’s modified Dulbecco’s medium (Gibco, Life Technologies Corp., CA, USA) with 20% FBS, 1% PS, and 0.8 mM LG.

### 4.2. Human Samples

Human peripheral blood mononuclear cells were obtained from healthy donors, and MCL and CLL cells were collected from patients after written informed consent in accordance with the Declaration of Helsinki.

### 4.3. CD37CAR Structure and Vector Construction

The CD37CAR structure backbone, which is composed of the anti-CD37 scFv based on the CAS-024 clone (variable light chain [VL]–linker–variable heavy-chain [VH])–12 amino acid IgG4 hinge (H)–CD28 transmembrane domain (TM)–CD28/CD40 intracellular domain (IC)–CD3ζ IC, was used as a template to modify in this study [15,25]. The two different scFv linkers, GSTSGSGKPGSGEGSTKG (Whitlow, 18aaL) [28] and GGGGSGGGGSGGGGSGGGGS (glycine–serine, GS4L), were designed and fused into the CD37CAR backbone using the NEBuilder HiFi DNA Assembly Cloning Kit (New England BioLabs, Ipswich, MA, USA). CAR constructs were then incorporated with a self-cleaving T2A skip sequence followed by a truncated epidermal growth factor receptor (tEGFR) to monitor CAR expression as previously described [15,28,39]. CAR genes were ligated into the LZRS-pBMN-Z vector and all sequences were validated by direct sequencing. Gammaretroviral particles were generated using the Phoenix-Ampho system (Orbigen, San Diego, CA, USA).

### 4.4. Generation of CD37CAR-T Cells

Mononuclear cells were isolated from the whole blood of healthy human volunteers using Lymphoprep (STEMCELL Technologies Inc., Vancouver, BC, Canada) and density-gradient centrifugation. CD3^+^ cells were further magnetically isolated following anti-human CD3 immunomagnetic bead incubation (Miltenyi Biotec, Bergisch Gladbach, Germany). The purified CD3^+^ cells were then activated with anti-CD3/CD28 Dynabeads (Invitrogen, Carlsbad, CA, USA) and cultured in RPMI 1640 medium (Gibco, Life Technologies Corp., CA, USA) containing 10% human serum, 0.8 mM LG, 1% PS, and 0.5 µM 2-mercaptoethanol (cytotoxic T-cell medium; CTL) and supplemented with 50 IU/mL recombinant human IL-2. On day 3, activated T-cells were transduced by spinoculation on a gammaretroviral particle pre-coated plate with recombinant human retronectin fragment (Retronectin, Takara Bio, Otsu, Japan) at 2100 rpm for 1 h at 32°C. On day 7, CAR^+^ T cells were enriched after incubating with biotin-conjugated anti-EGFR monoclonal antibody (R&D Systems, Minneapolis, MN, USA) and anti-biotin microbeads (Miltenyi Biotec, Bergisch Gladbach, Germany). Enriched CAR^+^ T cells were expanded by stimulating with anti-CD3/CD28 Dynabeads at a 1:1 ratio and cultured in CTL medium for 8–10 days before further use.

### 4.5. Fratricide Assay

Autologous CD3^+^ T cells were stained with CellTrace Violet (CTV) (Thermo Fisher Scientific, Waltham, MA, USA) and designed as target cells. The untransduced-T or CD37CAR-T cells were then cocultured with the target cells at a 1:1 ratio for 24 h. The live cell population (7-aminoactinomycin, AAD^−^) and the percentages of CTV+ cells were analyzed by flow cytometry. The CTV^+^ population was gated and the annexin V/propidium iodide (PI)^+^ cells were measured for apoptotic cell assessment.

### 4.6. T-Cell Proliferation and Cytokine Secretion Assays

Untransduced-T or CD37CAR-T cells were co-cultured with γ-irradiated Raji cells at a 1:2 E:T cell ratio in culture medium with or without IL-2 supplementation. After 24 h, the culture supernatant was collected for cytokine analysis. T-cell expansion was quantified using trypan blue staining to evaluate viable cells at the indicated time points. The IFN-γ, IL-2, and TNF-α concentrations were measured by enzyme-linked immunosorbent assay (ELISA) (BD Biosciences, Franklin Lakes, NJ, USA). An intracellular cytokine secretion assay was performed by stimulating untransduced-T or CD37CAR-T cells with Raji cells at the E:T ratio of 1:2 for 4 h. After 1.5 h, GolgiPlug^™^ Protein Transport Inhibitor (BD Biosciences, NJ, USA) was added. The cells were then fixed and permeabilized with Cytofix/Cytoperm^™^ (BD Biosciences). The stimulated cells were intracellular stained for IFN-γ, IL-2, Granzyme B, and perforin and analyzed by flow cytometry.

### 4.7. Intracellular Phospho-Flow Analysis

Untransduced-T or CD37CAR-T cells were stimulated with Raji cells at a 1:5 E:T ratio for 10 and 30 min. The cells were then harvested, spun down, and fixed with 2% formaldehyde at 37ºC for 10 min, permeabilized with cold 90% ethanol and incubated on ice for 30 min. The fixed cells were stained with primary phospho-specific antibodies including pZAP70 (Tyr319/Syk; Tyr352), pRelB (Ser552/D41B9), and phospho-NF-κB (pNF-κB p65; Ser536) (Cell Signaling Technology, Danvers, MA, USA) and secondary antibody with donkey anti-rabbit IgG (BioLegend, San Diego, CA, USA). Intracellular phosphorylated proteins were analyzed by flow cytometry.

### 4.8. CAR-T Cell Cytotoxicity Assay

Untransduced-T or CD37CAR-T cells were labeled with CTV and co-cultured with various target cell lines or primary tumor cell ratios (10:1, 5:1, 1:1, 1:5, and 1:10) for 24 h. The residual cells were collected and stained with 7-AAD and analyzed by flow cytometry. Target cell death was determined as CTV^−^/7AAD^+^ cell population. The percentage of specific cytolysis was calculated as [(target cell death − spontaneous cell death)/(100 − spontaneous cell death)] × 100. We performed prolonged co-culture assay to assess CAR-T cell cytotoxicity and persistence by incubating untransduced-T or CD37CAR-T cells with Raji/ffluc-GFP, MM1S/ffluc-GFP, or HUT78/ffluc-GFP at E:T ratios of 1:1 and/or 1:10 for 12 days without exogenous IL-2. The residual cells were measured by flow cytometry at designated time points to evaluate the proportions of effector and residual tumor cells.

### 4.9. Immunophenotypes

The monoclonal antibody-conjugated fluorophores in the experiments were CD3, CD8, CD45RA, CD62L, PD-1, CTLA-4, LAG3, TIM-3, granzyme B, perforin, 7-AAD, annexin V/PI (BioLegend, San Diego, CA, USA), CD37, and mCD45 (BD Biosciences, NJ, USA). All cell samples were analyzed by FACSAria™ Fusion (BD Biosciences, NJ, USA), and the data were analyzed by FlowJo software (https://www.flowjo.com, Tree Star, OR, USA).

### 4.10. Chronic Antigen Stimulation Assay

Untransduced-T or CD37CAR-T cells were repeatedly stimulated weekly with γ-irradiated Raji cells at a 1:1 ratio for three consecutive weeks in culture medium supplemented with IL-2. T-cell expansion and immunophenotypes for T-cell stemness and exhaustion were assessed weekly at pre- and post-stimulation.

### 4.11. Protein Structure Prediction and Validation

Modeling of the scFv region of the CD37 antibody was performed using the freely accessible ColabFold pipeline, which is an integration of an MMseqs2-based homology search server with AlphaFold2 that is a machine learning-based algorithm to accurately predict and generate the protein structures [40]. The amino acid sequence of CD37.18aaL CAR scFv with linker region of GSTSGSGKPGSGEGSTKG (241 amino acids) and CD37.GS4L CAR scFv with linker region of GGGGSGGGGSGGGGSGGGGS (243 amino acids) were input for protein modeling using default settings. The overall stereochemical quality of each modeled structure was assessed by template modeling (TM) score, predicted local distance difference test, predicted aligned error, and the Ramachandran plot implemented within the ColabFold platform. High-quality structure predictions were subjected for investigation of their binding affinity and dynamic behavior through protein–protein docking and molecular dynamic simulation, respectively.

### 4.12. Molecular Docking of Anti-CD37 Antibody scFv Toward Human CD37 Antigen

Molecular docking between the model structures (both CD37.18aaL and CD37.GS4L CAR scFv) and human CD37 receptor (retrieved from the Universal Protein Resource, UniProt: P11049; accession date: 1 October 2022) was performed using the ClusPro 2.0 web server with antibody mode to investigate their binding interaction toward its receptor. In the antibody mode, both CD37.18aaL and CD37.GS4L CAR scFv were defined as the receptor, whereas the CD37 receptor protein was considered as the ligand. All default docking parameters were executed during analysis. Among billions of sampling conformations, the most optimal model of antigen–antibody complexes was chosen based on the following contribution of the interaction energy between two proteins [41]. The complex structures of the 1000 lowest energies were clustered regarding the RMSD and were then refined to the final structure using energy minimization in ClusPro. Finally, the lowest energy docked complexes were analyzed to determine binding affinity (∆G) and dissociation constant (K_d_) values by PRODIGY (PROtein binDIng enerGY prediction) web-based service [42].

### 4.13. Molecular Dynamic Simulation of Anti-CD37 scFv

MD simulation of the protein structures has been used to mimic physiological conditions and determined their structural dynamic behaviors using GROMACS version 2022.4. All MD simulations of the predicted structures of CD37.18aaL and CD37.GS4L CAR scFv proteins were performed under the Amber FF99SB-ILDN force field. All systems were neutralized by counter ions and solvated in a modified TIP3P water model with 0.15M NaCl. Energy minimization was applied to remove steric clashes due to the unnatural overlap of any two non-bonding atoms in a protein structure until the maximum force was lower than 5000 kJ/mol/nm using the steepest descent algorithm. The equilibration of simulation systems was conducted with two phases of a position restraint of 1000 kJ/mol/nm applied to all heavy atoms of the proteins while allowing solvent molecules, ion molecules, and hydrogen atoms of proteins to move randomly. The first NVT simulation was conducted with a 2fs time step at a constant temperature of 300K for 500 picoseconds (ps), followed by NPT simulation under the constant pressure of 1 atm (1.013 bar) for 500 ps. All simulation systems were then subjected to 100 ns of an all-atoms MD simulation production phase in a temperature of 310K using a V-rescale thermostat with a coupling of 0.1 ps [43]. A pressure of 1 atm was held through an isotropic Berendsen barostat [44]. The LINCS algorithm constrained all hydrogen bond lengths [45]. The MD trajectories were written out every 10 ps and were then investigated via trajectory analysis modules implemented in the GROMACS suit. Structural analysis of MD simulations, including RMSD and RMSF of the structures’ backbone atoms, were plotted using the Grace package in Ubuntu 22.04.

### 4.14. Electrostatic Potential Analysis

To determine the electrostatic features of CD37.18aaL and CD37.GS4L scFv, the Coulombic EPS was computed by UCSF ChimeraX [46] using the *coulombic* command. The electrostatic potential on the molecular surfaces was visualized using the ChimeraX software (https://www.cgl.ucsf.edu/chimerax/, accessed on 1 May 2026). In addition, the total perturbed charge of the CAR scFvs was calculated using the tleap module of AmberTools 23 [47].

### 4.15. Murine Xenograft Models

Six- to eight-week-old male NOD/Shi-scid common-γ chain knockout (NSG) mice (CLEA Japan, Inc., Tokyo, Japan) were intravenously injected with Raji/ffluc-GFP (0.5 × 10^6^ cells) or MM1S/ffluc-GFP (3.0 × 10^6^ cells) via the tail vein as shown in the protocol Figure 4A and Figure 5A. The control tEGFR-transduced-T or CD37CAR-T (5.0 × 10^6^ cells) was then intravenously injected via the tail vein on day 7. Tumor volume was measured by bioluminescence imaging (BLI) (Caliper Life Science, Waltham, MA, USA) and the average radiances were quantified before T-cell transfer and then weekly thereafter to assess tumor progression. Survival was assessed based on death as the endpoint. Murine peripheral blood was obtained via the tail vein on day 10 after T-cell transfer. Erythrocyte lysis buffer (QIAGEN GmBH, Hilden, Germany) was used to remove the erythrocytes, and the residual leukocytes were then stained and analyzed by flow cytometry. Subsequently, the surviving mice were euthanized using carbon dioxide inhalation at a flow rate of 4 L/min on days 60 and 70 in Raji- and MM.1S mice models, respectively. The liver, spleen, and bone marrow were excised to assess T-cell persistence and immunophenotypes.

### 4.16. Statistical Analyses

All statistical analyses were performed using Prism version 10.4.2 software (GraphPad Software, La Jolla, CA, USA). Statistical significance was assessed using the student’s *t*-test, one-way ANOVA, or two-way ANOVA with appropriate Bonferroni or Tukey post-test corrections. The Kaplan–Meier method was evaluated through a log-rank (Mantel–Cox) test to determine the overall survival of the mice. The experimental data are presented as mean ± SEM. *p* values < 0.05 were considered significant. 

### 4.17. Study Approval

This study was approved by the Human Research Ethics Committee of the Faculty of Medicine, Prince of Songkla University, Thailand (REC.65-398-14-1). The mice were housed under specific pathogen-free conditions, and all murine experiments were approved by the Institutional Animal Care and Use Committee of Prince of Songkla University (Project license number MHESI 68014/457).

## Data Availability

The original contributions presented in this study are included in the article/supplementary material. Further inquiries can be directed to the corresponding author.

## Supplementary Materials

The following supporting information can be downloaded at: https://www.mdpi.com/article/10.3390/ijms27094112/s1.
