# Supplementary material for: Impact of Glycine–Serine Linker on Target Antigen Binding and Subsequent CD37CAR-T Performance
Source: Int J Mol Sci. 2026 May 4;27(9):4112. doi: 10.3390/ijms27094112 (PMC13163721; doi:10.3390/ijms27094112)
Supplement: Supplementary file 1 [file ijms-27-04112-s001.zip › Supplementary Figures 2026.04.04.pdf]

Supplemental Materials

Figure S1

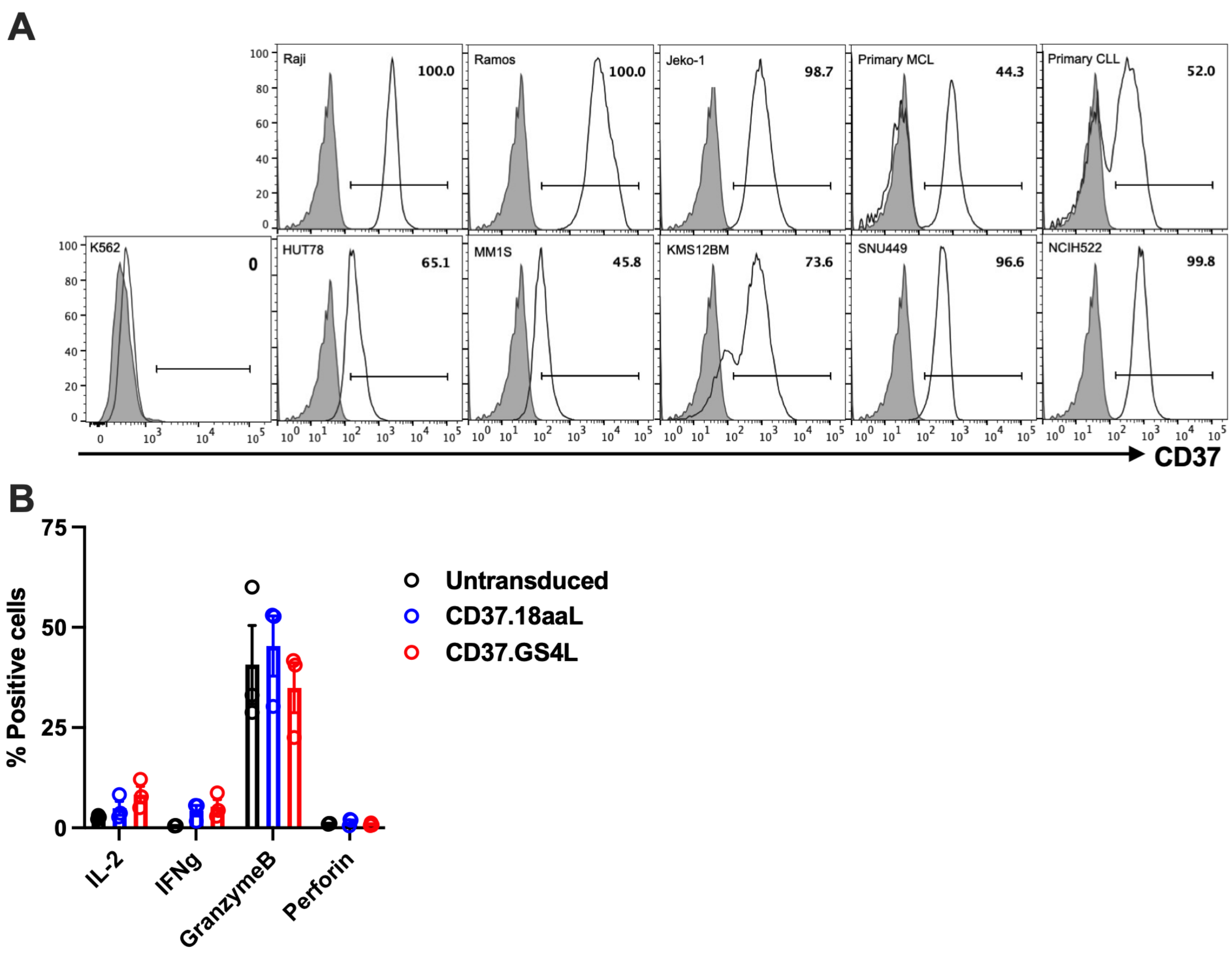

Figure S1 (A) Representative flow histograms of CD37 expression across various types of tumors: K562 (Chronic myeloid leukemia), Raji (Burkitt lymphoma), Ramos (Burkitt lymphoma), Jeko-1 (mantle cell lymphoma), primary chronic lymphocytic leukemia (CLL), primary mantel cell lymphoma (MCL), HUT78 (Sezary syndrome), MM.1S (multiple myeloma), KMS-12-BM (multiple myeloma) and solid tumor cell lines, SNU449 (hepatocellular carcinoma) and NCI-H522 (adenocarcinoma of lung). (B) Intracellular cytokine staining for IL-2, IFN-γ, granzyme B, and perforin. Untransduced-T or CD37CAR-T cells were stimulated with Raji at 1:2 ratio for 4 h then fixed and permeabilized. All data were pooled from three different donors and presented as mean ± SEM. One-way ANOVA; p = ns.

Figure S2

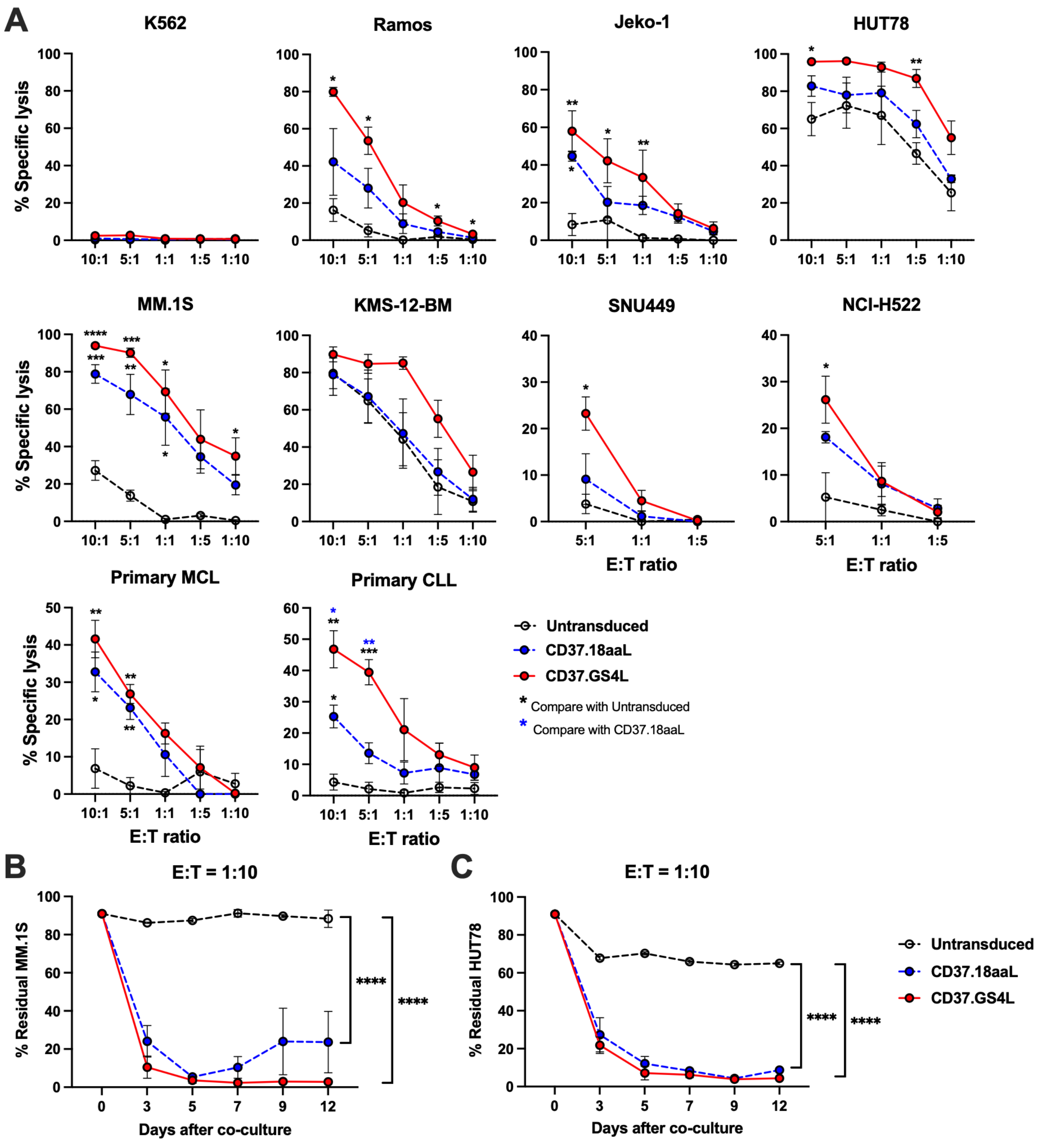

Figure S2 (A) Specific cytolysis assay of CD37CAR-T against various types of tumors: K562 (chronic myeloid leukemia; control), Raji (Burkitt lymphoma), Ramos (Burkitt lymphoma), Jeko-1 (mantle cell lymphoma), HUT78 (Sezary syndrome), MM.1S (multiple myeloma), KMS-12-BM (multiple myeloma), SNU449 (hepatocellular carcinoma), NCI-H522 (adenocarcinoma of lung), primary

chronic lymphocytic leukemia (CLL), and primary mantel cell lymphoma (MCL). CellTrace Violet-labeled untransduced-T or CD37CAR-T cells were incubated with tumor cells for 24 h at various E:T ratios. Dead cells were detected by 7-AAD Viability Staining Solution and flow cytometry. (B) Prolonged co-culture assay. Untransduced- or CD37CAR-T cells were co-cultured with MM.1S/ffluc-GFP or (C) HUT78/ffluc-GFP at E:T ratios of 1:10 for 12 days without IL-2 supplementation. The residual tumor cells were assessed by flow cytometry at indicated time points. Data were pooled from three different donors and shown as mean ± SEM. One-way ANOVA for (A) and two-way ANOVA for (B) and (C). *$p < 0.05$, **$p < 0.01$, ***$p < 0.001$, ****$p < 0.0001$.
